# Cremophor EL causes (pseudo-) non-linear pharmacokinetics of paclitaxel in patients

**DOI:** 10.1038/sj.bjc.6690696

**Published:** 1999-09

**Authors:** O van Tellingen, M T Huizing, V R Nannan Panday, J H M Schellens, W J Nooijen, J H Beijnen

**Affiliations:** Department of Clinical Chemistry, The Netherlands Cancer Institute (Antoni van Leeuwenhoek Huis), Plesmanlann 121, Amsterdam, 1066 CX, The Netherlands; Department of Pharmacy and Pharmacology, Slotervaart Hospital/The Netherlands Cancer Institute, Louwesweg 6, Amsterdam, 1066 EC, The Netherlands

**Keywords:** in vitro dialysis, plasma ultrafiltrate, free fraction, pharmacokinetic–pharmacodynamic relationship

## Abstract

The non-linear plasma pharmacokinetics of paclitaxel in patients has been well established, however, the exact underlying mechanism remains to be elucidated. We have previously shown that the non-linear plasma pharmacokinetics of paclitaxel in mice results from Cremophor EL. To investigate whether Cremophor EL also plays a role in the non-linear pharmacokinetics of paclitaxel in patients, we have established its pharmacokinetics in patients receiving paclitaxel by 3-, 24- or 96-h intravenous infusion. The pharmacokinetics of Cremophor EL itself was non-linear as the clearance (Cl) in the 3-h schedules was significantly lower than when using the longer 24- or 96-h infusions (Cl_175–3 h_ = 42.8 ± 24.9 ml h^−1^ m^−2^; Cl_175–24 h_ = 79.7 ± 24.3; *P* = 0.035 and Cl_135–3 h_ = 44.1 ± 21.8 ml h^−1^ m^−1^; Cl_140–96 h_ = 211.8 ± 32.0; *P* < 0.001). Consequently, the maximum plasma levels were much higher (0.62%) in the 3-h infusions than when using longer infusion durations. By using an in vitro equilibrium assay and determination in plasma ultrafiltrate we have established that the fraction of unbound paclitaxel in plasma is inversely related with the Cremophor EL level. Despite its relatively low molecular weight, no Cremophor EL was found in the ultrafiltrate fraction. Our results strongly suggest that entrapment of paclitaxel in plasma by Cremophor EL, probably by inclusion in micelles, is the cause of the apparent nonlinear plasma pharmacokinetics of paclitaxel. This mechanism of a (pseudo-)non-linearity contrasts previous postulations about saturable distribution and elimination kinetics and means that we must re-evaluate previous assumptions on pharmacokinetics–pharmacodynamics relationships. © 1999 Cancer Research Campaign


*British Journal of Cancer* (1999) **81**(2), 330–335
© 1999 Cancer Research Campaign
Article no. bjoc.1999.0696

### Cremophor EL causes (pseudo-) non-linear pharmacokinetics of paclitaxel in patients

**O van Tellingen[1], MT Huizing[2], VR Nannan Panday[2], JHM Schellens, WJ Nooijen[1] and JH Beijnen[2]**

[1]Department of Clinical Chemistry, The Netherlands Cancer Institute (Antoni van Leeuwenhoek Huis), Plesmanlann 121, 1066 CX Amsterdam, The Netherlands; [2]Department of Pharmacy and Pharmacology, Slotervaart Hospital/The Netherlands Cancer Institute, Louwesweg 6, 1066 EC Amsterdam, The Netherlands

**Summary** The non-linear plasma pharmacokinetics of paclitaxel in patients has been well established, however, the exact underlying mechanism remains to be elucidated. We have previously shown that the non-linear plasma pharmacokinetics of paclitaxel in mice results from Cremophor EL. To investigate whether Cremophor EL also plays a role in the non-linear pharmacokinetics of paclitaxel in patients, we have established its pharmacokinetics in patients receiving paclitaxel by 3-, 24- or 96-h intravenous infusion. The pharmacokinetics of Cremophor EL itself was non-linear as the clearance (Cl) in the 3-h schedules was significantly lower than when using the longer 24- or 96-h infusions ($Cl_{175–3\ h}$ = 42.8 ± 24.9 ml $h^{-1}$ $m^{-2}$; $Cl_{175–24\ h}$ = 79.7 ± 24.3; $P$ = 0.035 and $Cl_{135–3\ h}$ = 44.1 ± 21.8 ml $h^{-1}$ $m^{-1}$; $Cl_{140–96\ h}$ = 211.8 ± 32.0; $P$ < 0.001). Consequently, the maximum plasma levels were much higher (0.62%) in the 3-h infusions than when using longer infusion durations. By using an in vitro equilibrium assay and determination in plasma ultrafiltrate we have established that the fraction of unbound paclitaxel in plasma is inversely related with the Cremophor EL level. Despite its relatively low molecular weight, no Cremophor EL was found in the ultrafiltrate fraction. Our results strongly suggest that entrapment of paclitaxel in plasma by Cremophor EL, probably by inclusion in micelles, is the cause of the apparent nonlinear plasma pharmacokinetics of paclitaxel. This mechanism of a (pseudo-)non-linearity contrasts previous postulations about saturable distribution and elimination kinetics and means that we must re-evaluate previous assumptions on pharmacokinetics–pharmacodynamics relationships.

© 1999 Cancer Research Campaign

**Keywords:** in vitro dialysis; plasma ultrafiltrate; free fraction; pharmacokinetic–pharmacodynamic relationship

Paclitaxel (Taxol) is a potent anticancer drug with proven activity against a number of human solid tumours, including ovarian and breast, non-small-cell lung, and head and neck cancer (Huizing et al, 1995*a*; Rowinsky et al, 1995). Because of its poor water solubility the drug is formulated in a mixture of polyoxyethyleneglycerol triricinoleate 35 (Cremophor EL) and dehydrated ethanol USP (1:1, v/v; Taxol®). Prior to intravenous (i.v.) administration the drug solution is diluted 5- to 20-fold with saline or 5% dextrose. Patients receiving therapeutic dosages (e.g. 175 mg $m^{-2}$) of paclitaxel (Taxol) receive about 25 ml of Cremophor EL solvent. The non-linear pharmacokinetic behaviour of paclitaxel in patients has been well documented (Huizing et al, 1993; Sonnichsen et al, 1994; Gianni et al, 1995; Kearns et al, 1995; Huizing et al, 1997). Both a reduction in the clearance and an over proportional increase in peak plasma concentration ($C_{max}$) of paclitaxel are observed with increasing dosages indicative that both drug elimination and distribution were affected. We have recently shown that the non-linear pharmacokinetics of paclitaxel in mice results from Cremophor EL exclusively, since linear plasma pharmacokinetics occurred when the same dose levels of paclitaxel were administered in a Cremophor EL free drug formulation (Sparreboom et al, 1996*b*). In patients, administration of paclitaxel in a Cremophor EL free formulation to test if the pharmacokinetics becomes linear is not a feasible option. Indirectly, however, our observations support the idea that Cremophor EL may also be responsible for the non-linear pharmacokinetics in patients provided that the plasma levels of Cremophor EL are in the same range as those observed in mice.

In mice, the plasma concentrations of Cremophor EL as established by high-performance liquid chromatography (HPLC) ranged from 0.3 to 2.1% (Sparreboom et al, 1996*b*). Information on the pharmacokinetics of Cremophor EL in patients is limited. A previous paper using a bio-assay reported Cremophor EL levels up to 0.2% (Webster et al, 1993; Rischin et al, 1996). However, our preliminary information using the HPLC assay has revealed that the Cremophor EL levels are much higher (Sparreboom et al, 1996*b*; Van Tellingen et al, 1996). To address the issue if Cremophor EL can also be held responsible for the non-linear pharmacokinetics in patients, we have established the pharmacokinetic behaviour of this excipient in patients receiving 135 or 175 mg $m^{-2}$ of paclitaxel (Taxol) by 3- or 24-h infusions. Because we have previously suggested that alterations in the affinity of paclitaxel toward plasma components might provide an explanation for the nonlinear plasma pharmacokinetics (Sparreboom et al, 1996*b*), we have now used an in vitro equilibrium dialysis assay and determination of paclitaxel in plasma ultrafiltrate to assess the influence of Cremophor EL on plasma binding of paclitaxel.

*Received 12 November 1998*
*Revised 12 February 1999*
*Accepted 12 April 1999*

*Correspondence to:* O van Tellingen

### PATIENTS AND METHODS

### Patients

The patients included in this study received paclitaxel formulated in Cremophor EL:Ethanol 1:1 (v/v) diluted in saline, which was given

**Table 1** In vitro equilibrium dialysis assay.

| | | Falcon tube | | Insert tube | | |
|---|---|---|---|---|---|---|
| | | **Initial concentration** | **Equilibrium concentration** | **Initial concentration** | **Equilibrium concentration** | |
| Exp. 1 | Cremophor EL (%, v/v) | 0 | ND | 0 | ND | *n* = 3 |
| | Paclitaxel (ng/ml) | 1000 | 527 ± 52 | 1000 | 621 ± 37 | |
| Exp. 2 | Cremophor EL (%, v/v) | 0 | ND | 0.5 | ND | *n* = 2 |
| | Paclitaxel (ng/ml) | 1000 | 559 ± 20 | 1000 | 902 ± 6 | |
| Exp. 3 | Cremophor EL (%, v/v) | 0 | ND | 1.0 | ND | *n* = 3 |
| | Paclitaxel (ng/ml) | 1000 | 505 ± 22 | 1000 | 1051 ± 33 | |
| Exp. 4 | Cremophor EL (%, v/v) | 0 | ND | 2.0 | 1.68 | *n* = 3 |
| | Paclitaxel (ng/ml) | 1000 | 493 ± 23 | 1000 | 1180 ± 77 | |
| Control 1 | Cremophor EL (%, v/v) | 0 | ND | 0 | ND | *n* = 4 |
| | Paclitaxel (ng/ml) | 1000 | 474 ± 39 | 0 | 258 ± 35 | |
| Control 2 | Cremophor EL (%, v/v) | 2.0 | 1.88 ± 0.01 | 0 | <0.05 | *n* = 2 |
| | Paclitaxel (ng/ml) | 1000 | 678 ± 18 | 0 | <50 | |
| Control 3 | Cremophor EL (%, v/v) | 2.0 | 2.0 | No insert tube | *n* = 1 | |
| | Paclitaxel (ng/ml) | 1000 | 571 | | | |

ND = not determined. Initial concentrations indicate nominal concentrations of Cremophor EL and paclitaxel at the start of the experiment. At equilibrium (t = 64 h) the concentrations are measured; the results are given as equilibrium concentrations.

at a dose of 135 or 175 mg $m^{-2}$ by 3-h infusion or at a dose of 175 mg $m^{-2}$ in a 24-h infusion. Blood samples were collected by i.v. sampling from the arm opposite to the arm used for the infusion. This was performed in the 3-h infusion scheme before start, 1.5 h after the start, at the end of the infusion and at 6, 18, 30, 60 min and 2, 4, 8, 12, 24, 30 and 48 h after the end of the infusion. With the 24-h schedule the sampling was performed before the start, 3, 10 and 20 h after the start, at the end of the infusion and at 6, 18, 30, 60 min and 2, 4, 8, 12, 21, 30 and 48 h after the end of the infusion. Four to eight patients were included in each arm. Four additional patients received paclitaxel by a 96-h infusion administered through an IVAC i.v. administration set with low sorbing tubing (IVAC Corp., San Diego, CA, USA) and an IVEX-II vented filterset (0.22 µm; Millipore, Maksheim, France) was used for in-line filtration. Two patients received 140 mg $m^{-2}$ and the two others with liver metastases received 105 mg $m^{-2}$. Blood sampling was done before start, 8, 24, 48, 72 h after the start, at the end of the infusion and 2, 4, 10, 18 h after the end of the infusion. All patients had histologically proven ovarian or breast cancer and gave written informed consent. Further eligibility criteria included: (1) an Eastern Cooperative Oncology Group (ECOG) performance status ≤ 2, (2) adequate haematopoietic (absolute neutrophil count ≥ $2 \times 10^9$ $l^{-1}$, platelet count ≥ $100 \times 10^9$ $l^{-1}$), hepatic (total bilirubin ≤ 1.25 times the upper limit) and renal function (creatinine ≤ 1.5 times the upper limit), (3) no prior chemotherapy within 4 weeks before study entry (or 6 weeks in case of mitomycin C, high-dose carboplatin or nitrosoureas pretreatment) and (4) written informed consent. Dose preparations and premedication were standard and were described in detail earlier (Huizing et al, 1993).

### Cremophor EL determination

Cremophor EL levels in plasma have been quantified by an analytical method based on saponification of Cremophor EL, followed by extraction with chloroform and derivatization of the released fatty acid ricinoleic acid. The method has been described in detail previously (Sparreboom et al, 1996*a*), however, minor modifications to the method have been implemented to improve the safety of the assay and to reduce the chromatographic analysis time. In short, volumes of 50 µl of plasma were mixed with 50 µl of internal standard (2 mg $ml^{-1}$ margaric acid in methanol) and 200 µl of alcoholic potassium hydroxide USP. The samples were centrifuged for 5 min at 3000 ***g*** and the supernatants were transferred into a clean vial and incubated at 80°C for 30 min. Next, 200 µl of 1 M hydrochloric acid and 1 ml of chloroform were added and the vials were shaken vigorously for 10 min. After centrifugation, the aqueous layer was discarded and the organic fraction decanted into a clean glass tube and evaporated to dryness under vacuum using a Speed-Vac Plus SC210A (Savant, Faringdale, NY, USA) set at 43°C. The dried residues were dissolved by vortex-mixing in 500 µl of chloroform containing 10% (v/v) of oxalyl chloride and, next, incubated at 70°C for 30 min to form the fatty acid acyl-chlorides. Next, the tubes were dried by vacuum and 250 µl of 40 mM naphthyl amine in chloroform was rapidly added, followed by 50 µl of 5.6% (v/v) of triethylamine in chloroform. After vortex-mixing, the tubes were incubated at 37°C for 30 min, whereafter the organic solvent was removed by vacuum. The residue was redissolved in 1 ml of acetonitrile by sonication for 5 min. After adding 250 µl of water a volume of 20 µl was subjected to chromatography using a stainless steel column (10 × 4.6 mm) packed with 3 µm Microspher $C_{18}$ material kept at 40°C using a SPH-99 column oven (Spark, Emmen, The Netherlands). The mobile phase comprised a mixture of acetonitrile:10 mM potassium phosphate in water pH 7.0 (78:22; v/v) and was delivered at a flow rate of 1.5 ml $min^{-1}$. The column effluent was monitored using a UV detector operating at 280 nm. Peak height ratios of ricinoleic acid and internal standard were used for quantitative computations. Calibration curves ranged from 0.05% to 2.0% (v/v) of the plasma volume. The same procedure, without the saponification step in alcoholic potassium hydroxide, has been used to test the samples for the presence of ricinoleic acid, liberated by in vitro or in vivo degradation.

*© 1999 Cancer Research Campaign*

**Table 2** Pharmacokinetic parameters of Cremophor EL

| Taxol dose mg m$^{-2}$-h$^{-1}$ | Cremophor EL dose ml m$^{-2}$ | Patients | $C_{max}$ (%) | AUC (% h) | $t^1/_2$ (h) | CL (ml h$^{-1}$ m$^{-2}$) | $Vd_{ss}$ (l m$^{-2}$) |
|---|---|---|---|---|---|---|---|
| 175–3 | 14.6 | 1 | 0.70 | 46.6 | 58.5 | 31.3 | 2.54 |
| | | 2 | 0.70 | 61.1 | 73.1 | 23.9 | 2.71 |
| | | 3 | 0.57 | 36.3 | 49.1 | 40.2 | 2.80 |
| | | 4 | 0.58 | 49.9 | 68.2 | 29.2 | 2.95 |
| | | 5 | 0.62 | 30.4 | 39.6 | 47.9 | 2.62 |
| | | 6 | 0.67 | 41.7 | 53.7 | 35.0 | 2.66 |
| | | 7 | 0.45 | 14.3 | 26.3 | 101.8 | 3.78 |
| | | 8 | 0.63 | 43.6 | 55.6 | 33.4 | 2.87 |
| | | mean ± s.d. | 0.62 ± 0.08 | 40.6 ± 14.0 | 53.0 ± 15.0 | 42.8 ± 24.9 | 2.87 ± 0.39 |
| 175–24 | 14.6 | 9 | 0.42 | 31.6 | 46.6 | 46.0 | 2.76 |
| | | 10 | 0.37 | 18.7 | 35.4 | 78.0 | 3.60 |
| | | 11 | 0.29 | 14.6 | 36.1 | 99.9 | 4.75 |
| | | 12 | 0.38 | 15.4 | 22.6 | 94.7 | 2.41 |
| | | mean ± s.d. | 0.37 ± 0.05 | 19.7 ± 7.8 | 35.2 ± 9.8 | 79.7 ± 24.3 | 3.38 ± 1.04 |
| | | | | | $P = 0.059$[a] | $P = 0.035$[a] | $P = 0.40$[a] |
| 135–3 | 11.3 | 13 | 0.51 | 32.3 | 49.7 | 34.8 | 2.47 |
| | | 14 | 0.49 | 30.7 | 52.2 | 36.7 | 2.80 |
| | | 15 | 0.39 | 12.2 | 21.9 | 92.6 | 2.83 |
| | | 16 | 0.45 | 25.7 | 46.2 | 43.8 | 2.88 |
| | | 17 | 0.54 | 37.3 | 52.9 | 30.2 | 2.34 |
| | | 18 | 0.48 | 34.0 | 55.5 | 33.1 | 2.80 |
| | | 19 | 0.53 | 30.0 | 48.1 | 37.5 | 2.66 |
| | | mean ± s.d. | 0.48 ± 0.05 | 28.9 ± 8.1 | 46.6 ± 11.3 | 44.1 ± 21.8 | 2.68 ± 0.20 |
| | | | | | $P = 0.38$[a] | $P = 0.92$[a] | $P = 0.29$[a] |
| 140–96 | 11.7 | 20 | 0.083 | 6.5 | 12.7 | 179.8 | 5.52 |
| | | 21 | 0.063 | 4.8 | 13.9 | 243.8 | 4.88 |
| | | mean ± s.d. | | | 13.3 ± 0.8 | 211.8 ± 32.0 | 5.20 ± 0.45 |
| | | | | | $P = 0.005$[b] | $P < 0.001$[b] | $P < 0.001$[b] |
| 105–96 | 8.8 | 22 | 0.025 | 2.0 | 18.0 | 427.9 | 14.74 |
| | | 23 | 0.047 | 3.2 | 12.7 | 278.2 | 5.11 |

[a]Student's *t*-test relative to 175 mg m$^{-2}$ –3 h schedule; [b]Student's *t*-test relative to 135 mg m$^{-2}$ – 3 h schedule.

### Pharmacokinetic analyses

Pharmacokinetic analyses were performed using non-compartmental methods. The elimination constant (k) was calculated by linear regression of the final log-linear part of the concentration-time curves. The area under the plasma concentration time curves (AUC) was calculated using the linear trapezoidal rule from time = 0 until the last sampling point ($C_{last}$) and extrapolated to infinity using the formula $C_{last}$/k. The apparent Clearance (Cl = Dose/AUC) and the half-life ($t^1/_2$ = log 2/k) were estimated using classical equations. The area under the moment-time curve (AUMC) was also calculated by the linear trapezoidal rule with extrapolation to infinity (($C_{last} \times t_{last}$)/k + $C_{last}$/k$^2$), with $t_{last}$ being the time point at which $C_{last}$ was measured. The volume of distribution at steady-state ($Vd_{ss}$) was calculated using the equation: $Vd_{ss}$ = Dose ×AUMC/AUC$^2$ – Dose × Infusion time/(2 · AUC) The unpaired Student's *t*-test was used for statistical analysis with the computer program SPSS (version 6.1.3; SPSS Inc., Chicago, IL, USA). All reported *P*-values are based on two-sided tests of significance.

### In vitro equilibrium dialysis assay

All handlings were carried out under aseptic conditions in a laminar flow hood. Blank human plasma (Central Laboratory for the Blood Transfusion Services, Amsterdam, The Netherlands) was spiked with 1000 ng ml$^{-1}$ of paclitaxel (pure compound; Bristol-Myers Squibb Company, Princeton, NJ, USA) by dilution from a stock solution of 1 mg ml$^{-1}$ in methanol. Aliquots of 2.0 ml were pipetted into 15-ml polypropylene tubes (Falcon, Becton Dickinson Labware, Franklin Lakes, NJ, USA). Two-side open glass tubes (inside diameter: 8 mm) were fitted with a ZelluTrans Roth 2.0 V (molecular weight cut-off: 2000 Dalton) dialysis membrane (Carl Roth GmbH+Co, Karlsruhe, Germany) mounted on the bottom side. The effective dialysis surface was about 50 mm$^2$. Volumes of 300 µl of plasma were pipetted into these tubes, which were then inserted into the Falcon tubes with the membrane fully immersed in the plasma in the Falcon tube (Figure 1). The plasma in the insert tube contained 0, 0.5, 1 or 2% of Cremophor EL and 1000 ng ml$^{-1}$ of paclitaxel (Table 1: experiments 1–4 respectively). Insert tubes with blank plasma were used as a control to monitor whether equilibrium was reached. Insert tubes with blank human plasma placed in Falcon tubes containing 2.0 ml of 1000 ng ml$^{-1}$ of paclitaxel and 2% of Cremophor EL were used as control to check for passage of Cremophor EL through the membrane. Falcon tubes containing 1000 ng ml$^{-1}$ of paclitaxel and 2% of Cremophor EL, without insert tubes, were used as control to check the stability of these compounds during the incubation period. All tubes were incubated at 37°C for 64 h. Next, the plasma from the insert tube and the Falcon tube were separately stored until analysis. Paclitaxel levels were analysed using an HPLC method described previously (Huizing et al, 1995*b*).

*© 1999 Cancer Research Campaign*

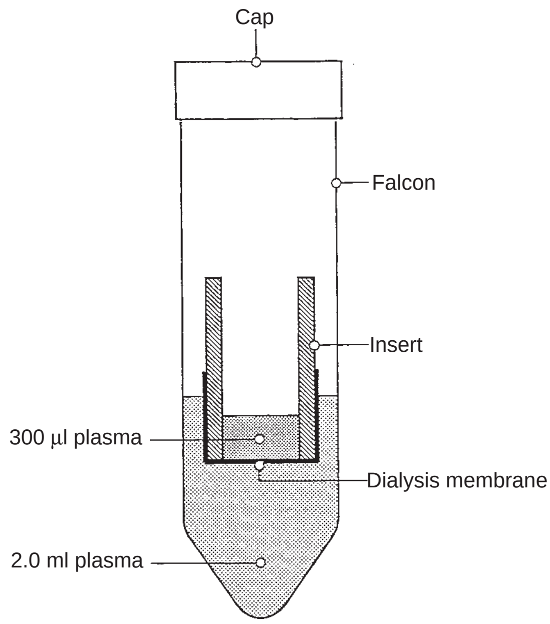

**Figure 1** Schematic setup of the *in vitro* equilibrium dialysis assay. Two side open glass 'insert' tubes were fitted with dialysis membrane and supplied with a volume of 300 µl of plasma. The tubes were inserted into a Falcon tube containing 2.0 ml of plasma and placed at 37°C for 64 h

### Preparation of plasma ultrafiltrate

Blank human plasma was spiked with 5000 ng ml$^{-1}$ of paclitaxel and 0, 0.5, 1 or 2% (v/v) of Cremophor EL. Plasma ultrafiltrate was prepared using Centrifree MPS-1 devices (Amicon, Danvers, MA, USA), 5 per Cremophor EL level, and pooled to obtain sufficient volume for analysis of paclitaxel and Cremophor EL.

### RESULTS

When 175 mg m$^{-2}$ of Taxol, corresponding to 14.6 ml m$^{-2}$ of Cremophor EL, was administered as 3-h infusion, the plasma concentration of Cremophor EL reached a maximum ($C_{max}$) of 0.62 ± 0.08% (v/v) of the plasma volume (Table 2 and Figure 2). At the 135 mg m$^{-2}$ dose level the peak level was proportionally lower at 0.48 ± 0.05%. After cessation of the infusion, the plasma levels started to decay without a clearly defined distribution phase. There was a considerable inter-patient variability and no statistically significant differences in $t^1/_2$ and Cl were found between the 175 and 135 mg m$^{-2}$ dose levels. However, a non-linear pharmacokinetic behaviour of Cremophor EL became evident when we take into account the results of the 24-h and 96-h infusion schedules. The Cl of Cremophor EL increased almost twofold ($P$ = 0.035) when the same dose (175 mg m$^{-2}$) of paclitaxel (Taxol) was given at this eightfold lower dose rate. Moreover, the clearance increased by fivefold ($P < 0.001$) if we compare the patient groups receiving 135 mg m$^{-2}$ by 3-h infusion and 140 mg m$^{-2}$ by 96-h infusion.

In all patients' samples, the plasma levels of ricinoleic acid, assayed by our Cremophor EL method with omission of the saponification step in alcoholic potassium hydroxide, remained below the lower limit of detection. Consequently, 'free' ricinoleic acid which might have been liberated from Cremophor EL by in vitro or in vivo degradation, was not present in plasma and did not interfere with the accuracy of the Cremophor EL determination.

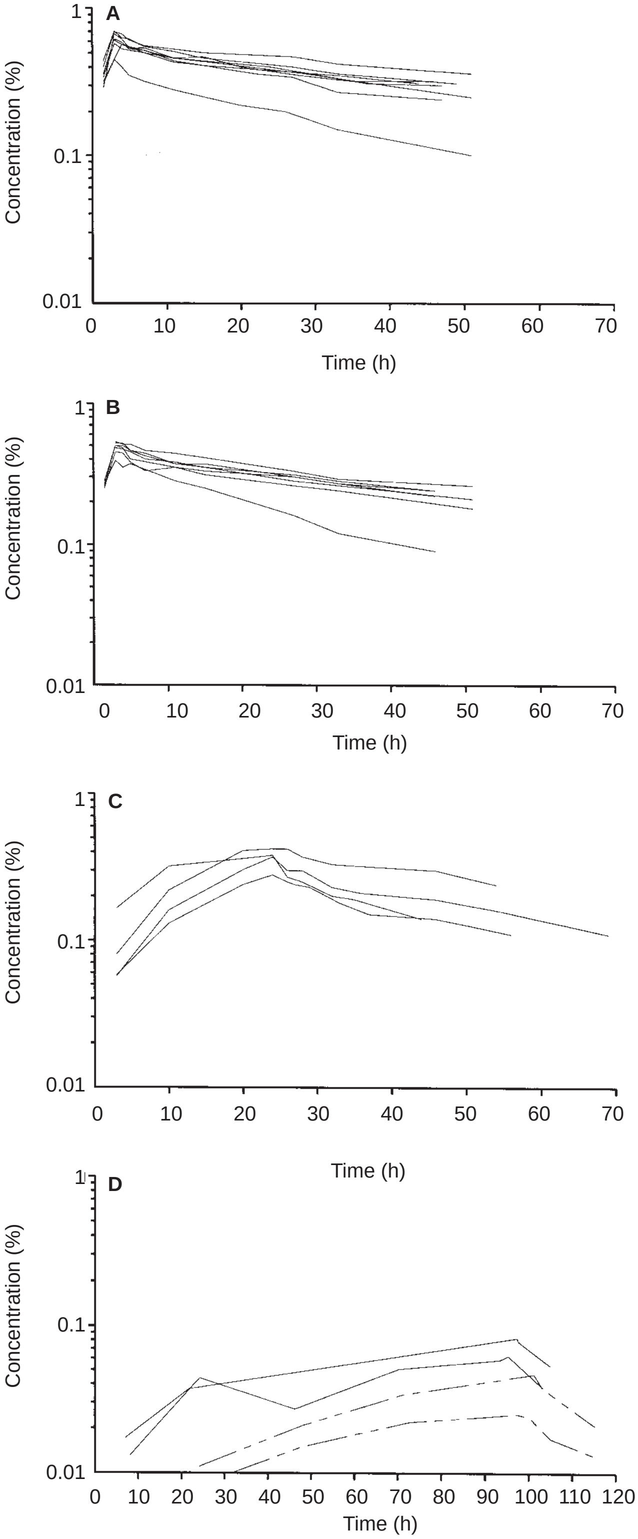

**Figure 2** The plasma concentration time profiles of Cremophor EL in individual patients receiving paclitaxel as 3-h infusion at a dose of 175 mg m$^{-2}$ (**A**) or 135 mg m$^{-2}$ (**B**), as 24-h infusion at a dose of 175 mg m$^{-2}$ (**C**) or as 96-h infusion (**D**) at a dose of 140 mg m$^{-2}$ (solid lines) or 105 mg m$^{-2}$ (dotted lines).

© 1999 Cancer Research Campaign

We have used an in vitro dialysis assay to study the impact of Cremophor EL on the binding of paclitaxel to plasma components. Given its low water solubility, the free paclitaxel levels in plasma were considered to be too low to use the classical setup for dialysis experiments using a protein-free acceptor buffer solution. The current setup with plasma on both sides of the membrane minimized the chances for losses due to absorption. Moreover, this setup easily allowed to estimate the differential plasma affinity as a function of increasing concentrations of Cremophor EL. Plasma from the same subject was used at both sides of the membrane. The dialysis membrane used in this study had a molecular weight molecular cut-off (MWCO) of 2000, because it needed to block the passage of Cremophor EL (approximate molecular weight = 3000) completely. However, as the molecular weight of paclitaxel (854) is also close to the cut-off value, passage through the membrane proceeds at a relatively low velocity and equilibrium between the two compartments was not yet reached within the 64-h incubation period (Control 1; Table 2). As can be seen from the specimen, which does not contain an insert tube (Control 3), over 40% of the paclitaxel was degraded within this period. The degradation by itself was not a problem, since we have analysed intact drug by HPLC, but it impeded the use of the much longer incubation periods required for full equilibration. However, although the current results may even somewhat underestimate the effect, it is clear that the presence of Cremophor EL increased the concentration of paclitaxel within the insert tube in a concentration dependent manner (Table 2). As expected, the Cremophor EL level at the opposite side of the membrane in the control experiment was below the lower limit of detection. However, in specimens containing 2.0% of Cremophor EL about 10% was recovered as ricinoleic acid, indicating (very slow) in vitro hydrolysis of Cremophor EL. To further validate the results of the in vitro dialysis assay we have also determined free paclitaxel by ultrafiltration. The free fraction of paclitaxel in plasma containing 0, 0.5, 1 and 2% (v/v) of Cremophor EL was 9.8%, 3.9%, 2.3% and 1.7%, respectively, thus showing a Cremophor EL dependent decrease in ultrafilterable paclitaxel. The concentration of Cremophor EL in the ultrafiltrate fraction was below the detection limit of the assay.

### DISCUSSION

This study shows that the plasma level of Cremophor EL after intravenous infusion of Taxol is sufficient to cause the non-linear plasma pharmacokinetics of paclitaxel in patients. Moreover, this nonlinearity is in fact a pseudo-non-linear pharmacokinetic behaviour. Non-linear pharmacokinetic behaviour of cytotoxic drugs is notorious, in particular because these drugs have a narrow therapeutic window and are usually administered at dosages close to the tolerated maximum. True non-linear pharmacokinetic behaviour is usually caused by saturation of drug elimination pathways (e.g. metabolizing enzymes) and the higher drug levels in plasma are a reflection of the higher amount of drug in the tissues. The non-linearity of paclitaxel caused by Cremophor EL is different. Our previous results in mice showed that the higher plasma level did not reflect higher drug tissue levels. Increasing the dose by five-fold from 2 to 10 mg $kg^{-1}$ resulted in a 30-fold higher $C_{max}$ of paclitaxel in plasma, whereas the tissue levels increased more linear by four- to sevenfold (Sparreboom et al, 1996*b*, 1996*c*). Based on this result, we hypothesized that the equilibrium in drug levels between plasma and tissues was shifted to plasma. Our in vitro equilibrium dialysis assay and plasma ultrafiltrate experiments using human plasma now clearly show that Cremophor EL, at clinically relevant concentrations, increases the affinity of paclitaxel to plasma. Consequently, the fraction of paclitaxel in plasma that is available for distribution and elimination ('free' paclitaxel) is a function of the Cremophor EL level. The nature of the interaction is not known, however, it is likely that Cremophor EL is capable of forming micelles, which can entrap paclitaxel. In aqueous solution the critical micellar concentration (CMC) of Cremophor EL is 0.009% (Jonkman-de Vries et al, 1996), although the actual CMC value in plasma may be higher due to the interference by other macro molecules. Further evidence that micelles are being formed results from the finding of very low levels of Cremophor EL in the ultrafiltered plasma fraction. Given the high Cremophor EL levels tested (up to 2%; v/v), it is very unlikely that all Cremophor EL is bound to plasma proteins. Free Cremophor EL, however, would have been able to freely pass the membrane (MW cut-off is 25 000). The absence of Cremophor EL in the ultrafiltrate indicates that Cremophor EL forms complexes (micelles), which are too large to pass the membrane. The finding of an increased affinity towards plasma containing Cremophor EL, together with the more or less linear increase in tissues observed in our study in mice, suggest that the distribution and elimination of 'free' paclitaxel are linear processes. The non-linear (e.g. more than proportional) increase in plasma AUC with dose is due to the measurement of 'total' drug levels. It is pseudo-non-linear pharmacokinetic behaviour because it does not relate to higher drug levels in tissues and will therefore not be accompanied by a corresponding more than proportional increase in pharmacodynamic effects. In fact, the higher affinity for plasma may lead to lower tissue levels as less drug is available for tissue distribution. However, since the ratio of the absolute amount of paclitaxel in tissues versus plasma is about 50:2 (*viz.* $Vd_{ss}$ of paclitaxel (Sonnichsen et al, 1994) versus the volume of plasma), even a large (e.g. two- or threefold) increase in plasma levels will only have a minor effect on the tissue levels. The concept of a Cremophor EL mediated increased affinity of paclitaxel to plasma provides a more logical explanation for the increasing $C_{max}$ of paclitaxel with increasing dose levels than the postulated saturability of paclitaxel transport into the tissues, which, although reported for other compounds previously (Gengo et al, 1984), is a very rare phenomenon.

Our results also demonstrate that the Cl of Cremophor EL is dependent on the dose-rate of the infusion. This nonlinear pharmacokinetic behaviour of Cremophor EL itself further augments the effects on paclitaxel and explains why the non-linear pharmacokinetics of paclitaxel is most evident when using short-term infusion schedules. The non-linearity of Cremophor EL may be due to the saturation of the enzyme(s) involved in its elimination. The $Vd_{ss}$ of Cremophor EL is very small and not (much) higher than the volume of the blood compartment. Although the pathway of elimination is currently unknown, it is possible that Cremophor EL may be partly degraded in the blood compartment. Given the long half-life such metabolic conversion in the blood occurs at a low rate and saturation of this metabolic site might easily occur given the high plasma levels. The two patients who demonstrated a higher elimination (*viz.* Table 1: patient no. 7 and no. 15) may have higher plasma concentrations of these metabolizing enzymes.

The non-linear pharmacokinetic behaviour of Cremophor EL, in particular when administered at lower dose rates, may have further consequences concerning the pharmacokinetics–pharmacodynamics relationships of paclitaxel. It has previously been shown that the toxic effect of paclitaxel (e.g. neutropenia) is correlated to

© 1999 Cancer Research Campaign the time at which the plasma levels of paclitaxel exceed 0.1 μM (Huizing et al, 1993). A further study by Gianni et al (1995) demonstrated that a threshold of 0.05 μM would be more appropriate. The latter study was initiated in part on the observation that when paclitaxel was administered as long-term (96-h) i.v. infusion, grade IV neutropenia was observed, although the threshold of 0.1 μM was not reached. The current work, however, shows that the Cremophor EL levels should be taken into account when making predictions based on paclitaxel plasma levels. The Cremophor EL levels of patients receiving the 96-h infusions were much lower than found in the 3- or 24-h schedules. As the effects of entrapment of paclitaxel in the plasma compartment will be less, the plasma levels of paclitaxel measured in the 96-h schedule will represent a higher fraction of 'free' drug. Consequently, the optimal value of the threshold level will vary with the schedule used for drug administration. A higher fraction of 'free' paclitaxel may also be anticipated when Taxol is given orally, since our study to test the feasibility of oral Taxol, given in combination with cyclosporin A, have shown that the plasma levels of Cremophor EL remain well below the lower limit of detection of the assay (Meerum Terwogt et al, 1998).

The plasma levels of Cremophor EL are substantially higher than reported previously in a paper utilizing a bio-assay (Rischin et al, 1996). This bio-assay is based on the property of Cremophor EL to increase the accumulation of daunorubicin in a leukaemia cell line (CEM/$VLB_{100}$), which overexpresses the multidrug resistance P-glycoprotein. The discrepancy between our chemical assay and the bio-assay may be due to the fact that Cremophor EL constitutes a relatively heterogenous group of polyoxyethylated triglyceride compounds and the bio-assay only recognizes the Cremophor EL fraction, which is responsible for the in vitro modulation of P-glycoprotein activity. A more or less selective clearance of this fraction would explain the lower levels observed by Rischin et al (1996), however, other factors affecting the intracellular daunorubicin accumulation cannot be excluded.

Our results are in line with those found using a colorimetric assay for Cremophor EL described very recently (Sparreboom et al, 1998*a*, 1998*b*). Although these authors studied the pharmacokinetics over a wide dose range (100–225 mg $m^{-2}$) using 3-h i.v. infusions of paclitaxel (Taxol), they concluded linear pharmacokinetic behaviour of Cremophor EL. However, their data does suggest non-linear kinetics of Cremophor EL as a twofold increase in the dose from 8.3 to 16.7 ml $m^{-2}$ resulted in a threefold increase in the AUC.

In conclusion, Cremophor EL is responsible for the non-linear plasma pharmacokinetics in humans. However, it is a pseudo-nonlinearity, since Cremophor EL increases the affinity of paclitaxel to plasma components in a dose-dependent manner and the resulting higher total drug levels in plasma do not reflect higher levels in tissues. Since the Cremophor EL levels depend strongly on the schedule used for drug administration, the plasma levels of Cremophor EL should be taken into account when making predictions of pharmacokinetics–pharmacodynamics relationships based on paclitaxel plasma levels.

### REFERENCES

Gengo FM, Schentag JJ and Jusko WJ (1984) Pharmacokinetics of capacity-limited distribution of methicillin in rabbits. *J Pharm Sci* **73**: 867–873

Gianni L, Kearns CM, Giani A, Capri G, Vigano L, Lacatelli A, Bonadonna G and Egorin MJ (1995) Nonlinear pharmacokinetics and metabolism of paclitaxel and its pharmacokinetic/pharmacodynamic relationships in humans. *J Clin Oncol* **13**: 180–190

Huizing MT, Keung AC, Rosing H, van der Kuij V, ten Bokkel Huinink WW, Mandjes IM, Dubbelman AC, Pinedo HM and Beijnen JH (1993) Pharmacokinetics of paclitaxel and metabolites in a randomized comparative study in platinum-pretreated ovarian cancer patients. *J Clin Oncol* **11**: 2127–2135

Huizing MT, Sewberath Misser VH, Pieters RC, ten Bokkel Huinink WW, Veenhof CH, Vermorken JB, Pinedo HM and Beijnen JH (1995*a*) Taxanes: a new class of antitumor agents. *Cancer Invest* **13**: 381–404

Huizing MT, Sparreboom A, Rosing H, Van Tellingen O, Pinedo HM and Beijnen JH (1995*b*) Quantification of paclitaxel metabolites in human plasma by high-performance liquid chromatography. *J Chromatogr B Biomed Appl* **674**: 261–268

Huizing MT, Giaccone G, Van Warmerdam LJC, Rosing H, Bakker PJM, Vermorken JB, Postmus PE, Van Zandwijk N, Koolen MGJ, ten Bokkel Huinink WW, Van der Vijgh WJF, Bierhorst FJ, Lai A, Dalesio O, Pinedo HM, Veenhof CHN and Beijnen JH (1997) Pharmacokinetics of paclitaxel and carboplatin in a dose-escalating and dose-sequencing study in patients with non-small-cell lung cancer. *J Clin Oncol* **15**: 317–329

Jonkman-de Vries JD, Flora KP, Bult A and Beijnen JH (1996) Pharmaceutical development of (investigational) anticancer agents – a review. *Drug Dev Indust Pharm* **22**: 475–494

Kearns CM, Gianni L and Egorin MJ (1995) Paclitaxel pharmacokinetics and pharmacodynamics. *Semin Oncol* **22**: 16–23

Meerum Terwogt JM, Beijnen JH, ten Bokkel Huinink WW, Rosing H and Schellens JHM (1998) Co-administration of cyclosporin enables oral therapy with paclitaxel. *Lancet* **352**: 285

Rischin D, Webster LK, Millward MJ, Linahan BM, Toner GC, Woollett AM, Morton CG and Bishop JF (1996) Cremophor pharmacokinetics in patients receiving 3-, 6-, and 24-hour infusions of paclitaxel. *J Natl Cancer Inst* **88**: 1297–1301

Rowinsky EK and Donehower RC (1995) Paclitaxel (Taxol). *N Engl J Med* **332**: 1004–1014

Sonnichsen DS, Hurwitz CA, Pratt CB, Shuster JJ and Relling MV (1994) Saturable pharmacokinetics and paclitaxel pharmacodynamics in children with solid tumors. *J Clin Oncol* **12**: 532–538

Sparreboom A, Van Tellingen O, Huizing MT, Nooijen WJ and Beijnen JH (1996*a*) Determination of polyoxyethyleneglycerol triricinoleate 35 (Cremophor EL) in plasma by pre-column derivatization and reversed-phase high-performance liquid chromatography. *J Chromatogr* **681**: 355–361

Sparreboom A, Van Tellingen O, Nooijen WJ and Beijnen JH (1996*b*) Nonlinear pharmacokinetics of paclitaxel in mice results from the pharmaceutical vehicle Cremophor EL. *Cancer Res* **56**: 2112–2115

Sparreboom A, Van Tellingen O, Nooijen WJ and Beijnen JH (1996*c*) Tissue distribution, metabolism and excretion of paclitaxel in mice. *Anticancer Drugs* **7**: 78–86

Sparreboom A, Loos WJ, Verweij J, Devos AI, Van der Burg MEL, Stoter G and Nooter K (1998*a*) Quantitation of Cremophor EL in human plasma samples using a colorimetric dye-binding microassay. *Anal Biochem* **255**: 171–175

Sparreboom A, Verweij J, Van der Burg MEL, Loos WJ, Brouwer E, Vigano L, Lacatelli A, de Vos AI, Nooter K, Stoter G and Gianni L (1998*b*) Disposition of Cremophor EL in humans limits the potential for modulation of the multidrug resistance phenotype in vivo. *Clin Cancer Res* **4**: 1937–1942

Van Tellingen O, Sparreboom A, Huizing MT, Nooijen WJ and Beijnen JH (1996) The analysis and preliminary pharmacokinetics of Cremophor EL in human plasma. *Ann Oncol* **7**: 92

Webster L, Linsenmeyer M, Millward M, Morton C, Bishop J and Woodcock D (1993) Measurement of cremophor EL following taxol: plasma levels sufficient to reverse drug exclusion mediated by the multidrug-resistant phenotype. *J Natl Cancer Inst* **85**: 1685–1690

*© 1999 Cancer Research Campaign*
